# Providing Doctors With High-Quality Information: An Updated Evaluation of Web-Based Point-of-Care Information Summaries

**DOI:** 10.2196/jmir.5234

**Published:** 2016-01-19

**Authors:** Koren Hyogene Kwag, Marien González-Lorenzo, Rita Banzi, Stefanos Bonovas, Lorenzo Moja

**Affiliations:** ^1^ Clinical Epidemiology Unit IRCCS Galeazzi Orthopedic Institute Milan Italy; ^2^ Laboratory of Drug Regulatory Policies IRCCS Mario Negri Institute for Pharmacological Research Milan Italy; ^3^ Humanitas Clinical and Research Center Milan Italy; ^4^ Department of Biomedical Sciences for Health University of Milan Milan Italy

**Keywords:** point-of-care summaries, internet information, evidence-based medicine, information science

## Abstract

**Background:**

The complexity of modern practice requires health professionals to be active information-seekers.

**Objective:**

Our aim was to review the quality and progress of point-of-care information summaries—Web-based medical compendia that are specifically designed to deliver pre-digested, rapidly accessible, comprehensive, and periodically updated information to health care providers. We aimed to evaluate product claims of being evidence-based.

**Methods:**

We updated our previous evaluations by searching Medline, Google, librarian association websites, and conference proceedings from August 2012 to December 2014. We included Web-based, regularly updated point-of-care information summaries with claims of being evidence-based. We extracted data on the general characteristics and content presentation of products, and we quantitatively assessed their breadth of disease coverage, editorial quality, and evidence-based methodology. We assessed potential relationships between these dimensions and compared them with our 2008 assessment.

**Results:**

We screened 58 products; 26 met our inclusion criteria. Nearly a quarter (6/26, 23%) were newly identified in 2014. We accessed and analyzed 23 products for content presentation and quantitative dimensions. Most summaries were developed by major publishers in the United States and the United Kingdom; no products derived from low- and middle-income countries. The main target audience remained physicians, although nurses and physiotherapists were increasingly represented. Best Practice, Dynamed, and UptoDate scored the highest across all dimensions. The majority of products did not excel across all dimensions: we found only a moderate positive correlation between editorial quality and evidence-based methodology (*r*=.41, *P*=.0496). However, all dimensions improved from 2008: editorial quality (*P*=.01), evidence-based methodology (*P*=.015), and volume of diseases and medical conditions (*P*<.001).

**Conclusions:**

Medical and scientific publishers are investing substantial resources towards the development and maintenance of point-of-care summaries. The number of these products has increased since 2008 along with their quality. Best Practice, Dynamed, and UptoDate scored the highest across all dimensions, while others that were marketed as evidence-based were less reliable. Individuals and institutions should regularly assess the value of point-of-care summaries as their quality changes rapidly over time.

## Introduction

Pressed for time and obliged to navigate ever-expanding medical literature, doctors are increasingly relying on online information tools to accelerate the search process without compromising the reliability and quality of information retrieved. Point-of-care information summaries offer predigested syntheses of medical research intended to be used when the patient and physician interact (ie, point-of-care) [1]. Web-based point-of-care summaries provide user-friendly interfaces that may improve the retrieval, synthesis, organization, and application of evidence-based content in clinical practice [2,3].

The medical information technology market parallels the efforts by national health systems to streamline clinical workflow and align clinicians’ behavior with best practice strategies. Point-of-care summaries play a central role: they increasingly form the knowledge basis of complex information systems, such as computerized physician order entry and computer decision support systems [3-5]. In the United States, the Health Information Technology for Economic and Clinical Health (HITECH) Act requires clinicians and hospitals to integrate electronic health records (EHRs) with clinical decision support rules relevant to a specialty or to high-priority hospital conditions, such as drugs and diagnostic test ordering [6]. In Europe, the integration of point-of-care summaries into the workflow of the prescribers is under scrutiny in several countries [7-10].

As point-of-care information summaries gain ground in the culture of medical practice as stand-alone products or integrated with other systems, their validity must be assessed against marketing claims that they are evidence-based. This review examines the quality of Web-based point-of-care information summaries and their development and progress since 2008.

## Methods

### Inclusion and Exclusion Criteria

As this is an update of analyses done in 2008 [11] and 2012 [12], detailed methods and operational definitions can be found in the original publication [11]. Briefly, we defined point-of-care information summaries as “Web-based medical compendia specifically designed to deliver predigested, rapidly accessible, comprehensive, periodically updated, and evidence-based information (and possibly also guidance) to clinicians.” To be included in this review, a product had to be an online-delivered tertiary publication (summary) that is regularly updated, claims to provide evidence-based information to physicians and other professionals, and is intended for use at the bedside. We considered summaries, regardless of their content development status, number of years on the market, clinical focus or specialty, type of access, or charging agreements. We excluded other online information resources such as guideline databases, meta-lists and search engines, literature surveillance alerting systems, online books, and journal articles (ie, primary and secondary literature). Our analysis was limited to products in the English language.

### Search Strategy

To identify the point-of-care information summaries, we re-examined the eligibility of all products that were included or excluded in the 2008 and 2012 analyses. To find new summaries, we searched Medline from August 2012 to December 2014 with the following terms: ((“Evidence-Based Medicine”[Mesh]) AND (“Information Storage and Retrieval”[Mesh])) AND ((“Online Systems”[Mesh]) OR (“Point-of-Care Systems”[Mesh])). We scanned the references of the papers retrieved and used the Google search engine to identify additional products that may not have been reported in the medical literature. We explored various publisher and librarian association websites (ie, Council of Science Editors, the World Association of Medical Editors, the European Association for Health Information and Libraries, and the American Medical Informatics Association) [13-16], and the 2014 conference proceedings from the Medical Library Association Meeting and Exhibition [17].

### Identification of Point-of-Care Information Summaries

One reviewer examined the search results, screened the titles and abstracts of papers identified through Medline, and evaluated the eligibility of products integrating additional information found on product websites. If there was doubt about the inclusion of a product, all authors discussed the eligibility until a consensus was reached. We recorded the reasons for exclusion.

### Data Extraction and Analysis

One reviewer extracted information on the general features of each point-of-care information summary. Products that could not be accessed (ie, no subscription available at our institution, no free-trial option, and no response from product representatives to our emails requesting access) were excluded. One reviewer collected data on the general characteristics of products and their content presentation for qualitative (descriptive) evaluation, along with information about the editorial quality, evidence-based methodology, and content volume (breadth of diseases and medical conditions covered) for empirical quantitative analysis. A second reviewer checked the extractions.

### Qualitative Evaluation

For each summary included, we collected the following general details: country of development, year of release, vendor or publisher, marketing claims, format (eg, tablets, mobile devices), access and subscription options, annual costs, and targeted audience. Since the 2012 analysis, we have introduced an additional component: ability to be integrated into an EHR system. This entails the capacity to access information from the point-of-care summary directly through the EHR interface. For example, when a physician clicks on a condition written in the patient record, the physician is directed to a new screen detailing disease information and treatment options. A point-of-care summary search tool may be additionally available on the EHR interface to make free-text and *International Classification of Diseases* (ICD)-10 code searches.

Content presentation was analyzed in summaries that we accessed. We examined the different outputs (eg, key point summary, paragraphs, question and answers, book chapter-like summary, clinical pathway, clinical scenario), use of formal ontology, flexibility, and reporting of references (with or without general or specific citations). We also assessed products’ adoption of an intent to recommend, use of a formal strength of recommendation system, as well as the availability of continuing medical education programs or credits, other education materials (eg, lessons on statistical analysis or evidence-based methodology), and patient handouts.

### Quantitative Analysis

Two reviewers extracted information about three key dimensions: quality of the editorial process, quality of the evidence-based approach to content development (ie, evidence-based methodology), and volume or breadth of the medical conditions covered. We described products quantitatively using three separate scores that covered components relevant to each dimension. Disagreements were resolved by discussion between the reviewers. A third author was consulted for any unsolved discordances. All Web pages providing useful data were saved and stored in an electronic archive. When information about a particular component (eg, commercial support or critical appraisal) was unclear or could not be found, we contacted publishers by email requesting additional information and clarification of contents. All emails were stored in an electronic archive.

#### Editorial Quality

We adopted the following indicators of transparency to evaluate the methodological quality of the editorial process: authorship (reporting of authors for each summary), reviewing (implementation of a formal, structured peer-review process), updating (whether or not summaries had been revised or updated in the previous 2 years), conflicts of interest (disclosure of contributing authors’ conflict of interest), and commercial support for content development. For this last component, we assigned three points if commercial support was not accepted, one point if commercial support was accepted and reported, and no points if the product developer did not present sufficient information for us to make a judgement. For the remaining items, we assigned three points if the component was judged as “adequate,” one point if “unclear,” and none if “not adequate” or “not reported.” We arbitrarily decided to award three points instead of two for the adequate fulfillment of a criteria in order to give more weight to transparent and accountable reporting, and increase variability within the sample.

In the 2008 and 2012 reviews, we assessed the authorship, authors’ conflicts of interest, and updating of products based on the editorial policy statements. If the information provided was insufficient to make an accurate evaluation, we referred to a nonrandom selection of sections (often referred as topics) to assess the dimensions. In the effort to minimize bias between reporting and implementation in the 2014 analysis, we evaluated these dimensions through a random sample of topics. We randomly selected ten blocks or categories of diseases from ICD-10 [18]. If any product did not cover one of the medical conditions identified in a block, we randomly selected another block from ICD-10. In each topic, we checked the reporting of authors as well as any potential conflict of interest. For updating, topics were considered up-to-date if they had been reviewed or revised within the last 2 years (January 2013 to January 2015). The 2-year time frame was determined based on the average time to changes in evidence that are sufficiently important to require the updating of systematic reviews [19]. Products with eight or more topics updated in the last 2 years were assigned 3 points towards the total editorial quality score. Products with three or less topics updated within that period were assigned no points. Other products with four to seven updated topics were assigned 1 point as well as those that did not consistently provide dates on the articles.

#### Evidence-Based Methodology

The following components were used to evaluate the strength of the evidence-based methodology for content development: implementation of a literature search or surveillance strategy to identify current information, cumulative versus discretionary approach (prioritization of systematic reviews over other evidence sources), critical appraisal, formal grading of evidence, and citation of expert opinions (separation of expert opinions from other evidence sources in summaries). Three points were assigned if the component was judged “adequate,” one if considered “unclear,” and none if “not adequate” or “not reported.”

#### Volume (Breadth of Diseases Covered)

As it was not feasible to count the total number of diseases and medical conditions covered in each product, we estimated the comprehensiveness of disease coverage by verifying the presence or absence of a random sample of diseases from the ICD-10 [18]. We randomly selected four chapters: Chapter IV—Endocrine, nutritional and metabolic diseases, VII—Diseases of the eye and adnexa, XII—Diseases of the skin and subcutaneous tissue, and XV—Pregnancy, childbirth and the puerperium. These chapters comprised a total of 35 blocks or categories of diseases or medical conditions. If a point-of-care information summary discussed at least one disease specified within a block, the product was assigned 1 point towards a maximum of 35 total points for volume. We then converted the volume scores into percentages, where 35 points correspond to 100% coverage.

Multimedia Appendix 1 summarizes in a flow diagram the methods used to evaluate products.

### Analysis

Volume and quality indicator scores are presented with medians and interquartile ranges (IQR). Point-of-care information summaries were ranked on the basis of (1) editorial quality, (2) the use of an evidence-based approach, and (3) the volume of diseases covered based on a random sample of ICD-10 chapters. Correlations between these three dimensions were assessed by Spearman rank correlation coefficients and their respective *P* values. Changes in the strength of the products from 2008-2014 were assessed using the matched pairs Wilcoxon signed-rank test. For hypothesis testing, a probability of <.05 was considered statistically significant. All statistical tests were two-sided. Stata software was used for statistical analyses.

## Results

**Figure 1:**
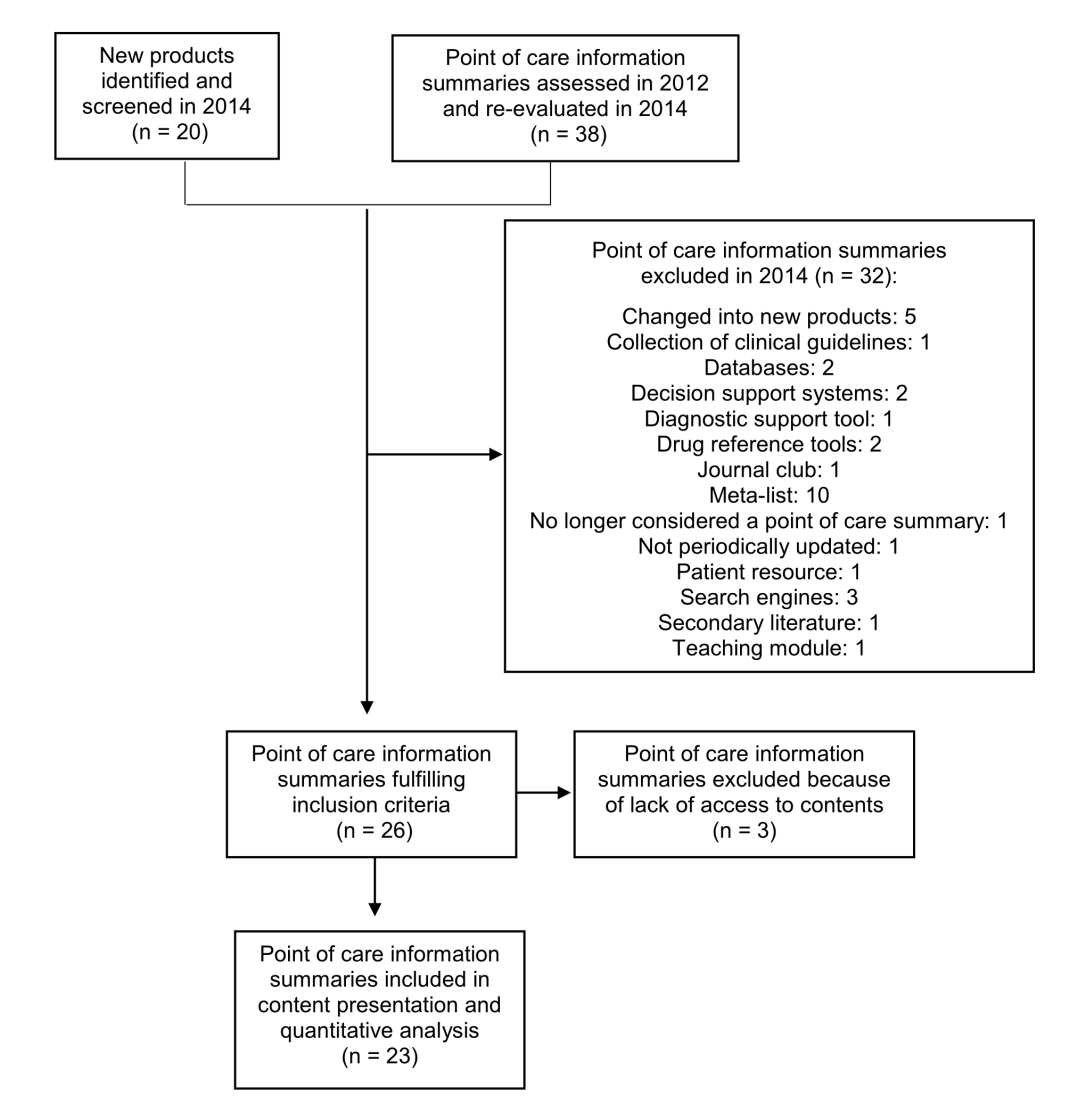
Flow diagram of point-of-care information summaries included in the review.

The search strategy identified 58 products for potential inclusion. After screening, 26 fulfilled our inclusion criteria. Sixteen of these were previously included in the 2008 and 2012 reviews (5 Minute Consult, BestBets, Clin-eGuide, Dynamed, EBM Guidelines, Essential Evidence Topics, eTG Complete, GP Notebook, Map of Medicine, Micromedex, Mosby’s Nursing Consult, Nursing Reference Center, PEPID, Rehabilitation Reference Center, UpToDate, and Zynx Evidence). Four products changed into a new product since 2012 (ACP Smart Medicine formerly ACP Pier, Best Practice formerly Clinical Evidence, Clinical Key formerly First Consult, Medscape Drug and Diseases Reference formerly Emedicine). Six products were newly identified in this review (Clinical Access, Cochrane Clinical Answers, Decision Support in Medicine, NICE Pathways, PEMSoft, and Prodigy). Prodigy, which is connected with Clinical Knowledge Summaries (CKS), was considered a new product since CKS was discontinued for some time and only in 2012 was restarted. Figure 1 shows the flow diagram for the selection of point-of-care information summaries in the review.

In order to access the 26 products, we registered for free-trial access online whenever available or contacted the publishers directly requesting temporary access to perform the evaluation. We did not receive a response from the publishers of three products (Clin-eGuide, Mosby’s Nursing Consult, and Zynx Evidence), which were prevented from further evaluation. A total of 23 products were included in the content presentation and quantitative analysis.

### Qualitative Evaluation

General features are summarized in Multimedia Appendix 2. Most of the 26 products were developed by major publishers in the United States (n=12) and United Kingdom (n=8), while others came from the Netherlands (n=4), Finland (n=1), and Australia (n=1). A minority was open access (19%), while most were fee-based (81%) with a median individual subscription price of €244.4 (US$265, £169.52). Regarding their electronic compatibility, over a quarter (7/26, 27%) of products were Web-based only, as others could also be opened on mobile devices. Most products targeted a general audience of health professionals (18/26, 70%), but some were advertised for specific groups such as medical specialists (1/26, 4%), general practitioners (2/26, 8%), nurses (2/26, 8%), emergency medicine doctors (1/26, 4%), pediatricians (1/26, 4%), and rehabilitation professionals (1/26, 4%). Sixteen products out of 26 (62%) could be integrated into EHRs.

Multimedia Appendix 3 presents details of the summary content presentation of the 23 products we could fully evaluate. Products displayed their content in a variety of formats: key point summary, questions and answers, book chapter-like summaries, and clinical pathways (flow charts). Most had a formal ontology for organizing diseases and medical conditions (20/23, 87%) as well as flexible navigation of topic contents (19/23, 83%). Although many products adopted an intent to recommend approach (17/23, 73%), under a third (7/23, 30%) used a formal strength of recommendation system: Grades of Recommendation, Assessment, Development and Evaluation (GRADE) approach [20], the Strength of Recommendation taxonomy (SORT) by the American Academy of Family Physicians [21], or individual systems developed for the product. Just under a half (11/23, 48%) of products awarded continuing medical education credits for searches or featured other programs for continuing medical education. Patient education materials and handouts were available in nearly a third (7/23, 30%) of products, and only a few (4/23, 17%) offered additional educational materials for clinicians such as evidence-based medicine and critical appraisal methodology, lessons on cultural competencies, laboratory manuals, and practice resources.

### Quantitative Analysis

**Figure 2:**
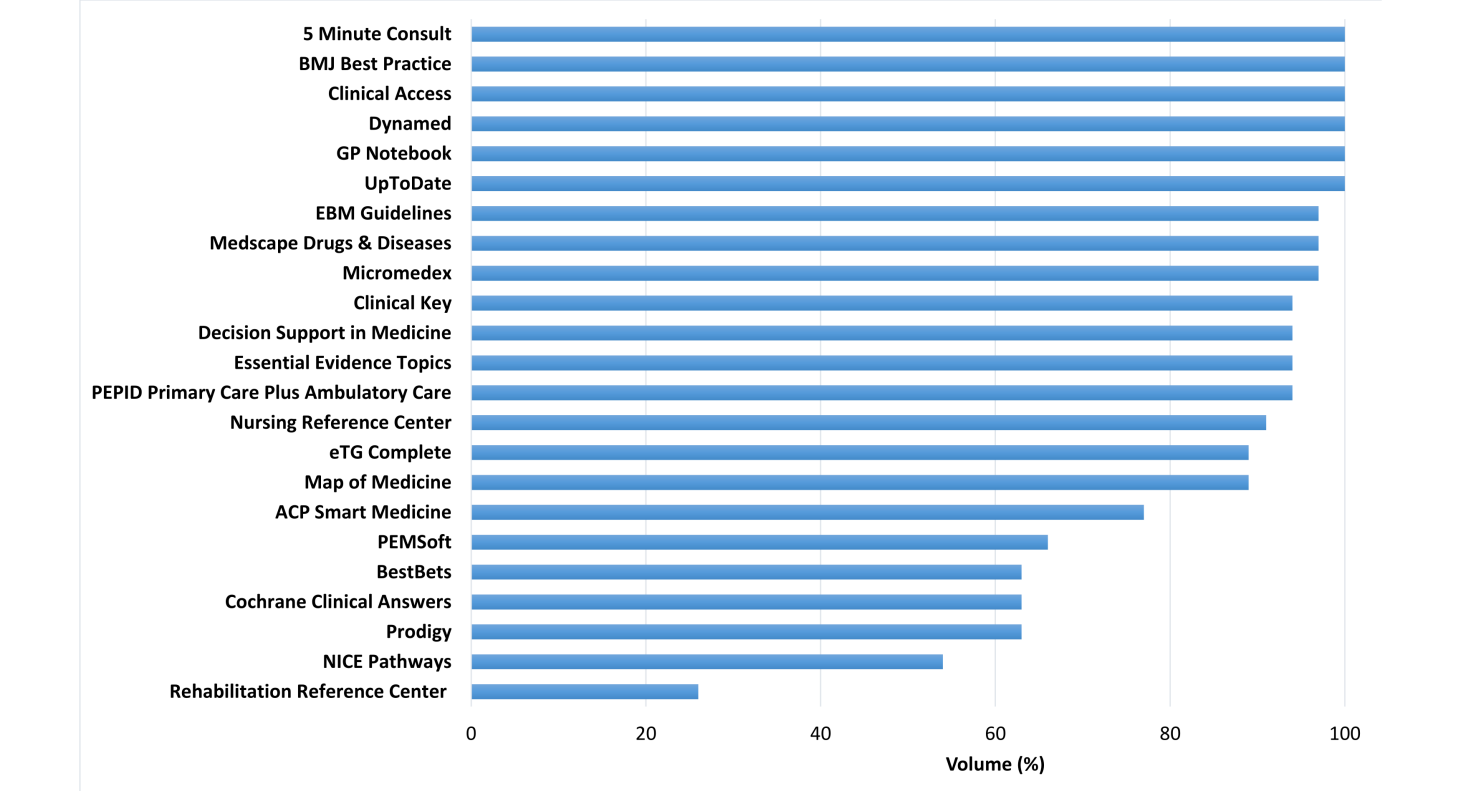
Estimated volume (breadth) of diseases and medical conditions covered by point-of-care information summaries (based on 4 randomly selected chapters of the ICD-10 classification system).

Figure 2 shows the rank of products based on volume. Disease coverage varied widely: the median volume or coverage of medical conditions was 94% (IQR, 66-100%). The most comprehensive products providing at least one condition per disease category in the four ICD-10 chapters were 5 Minute Consult, Best Practice, Clinical Access, Dynamed, GP Notebook, and UpToDate.

Editorial quality and evidence-based methodology are summarized in Multimedia Appendices 4 and 5; the median scores were 12 (IQR 6-13) and 11 (IQR 4-15), respectively, on a 15-point scale. Five products (ACP Smart Medicine, BMJ Best Practice, Dynamed, Essential Evidence Topics, and UpToDate) received the maximum score for editorial quality. Six (ACP Smart Medicine, BestBets, BMJ Best Practice, Dynamed, EBM Guidelines, and UpToDate) received the maximum score for evidence-based methodology.

**Figure 3:**
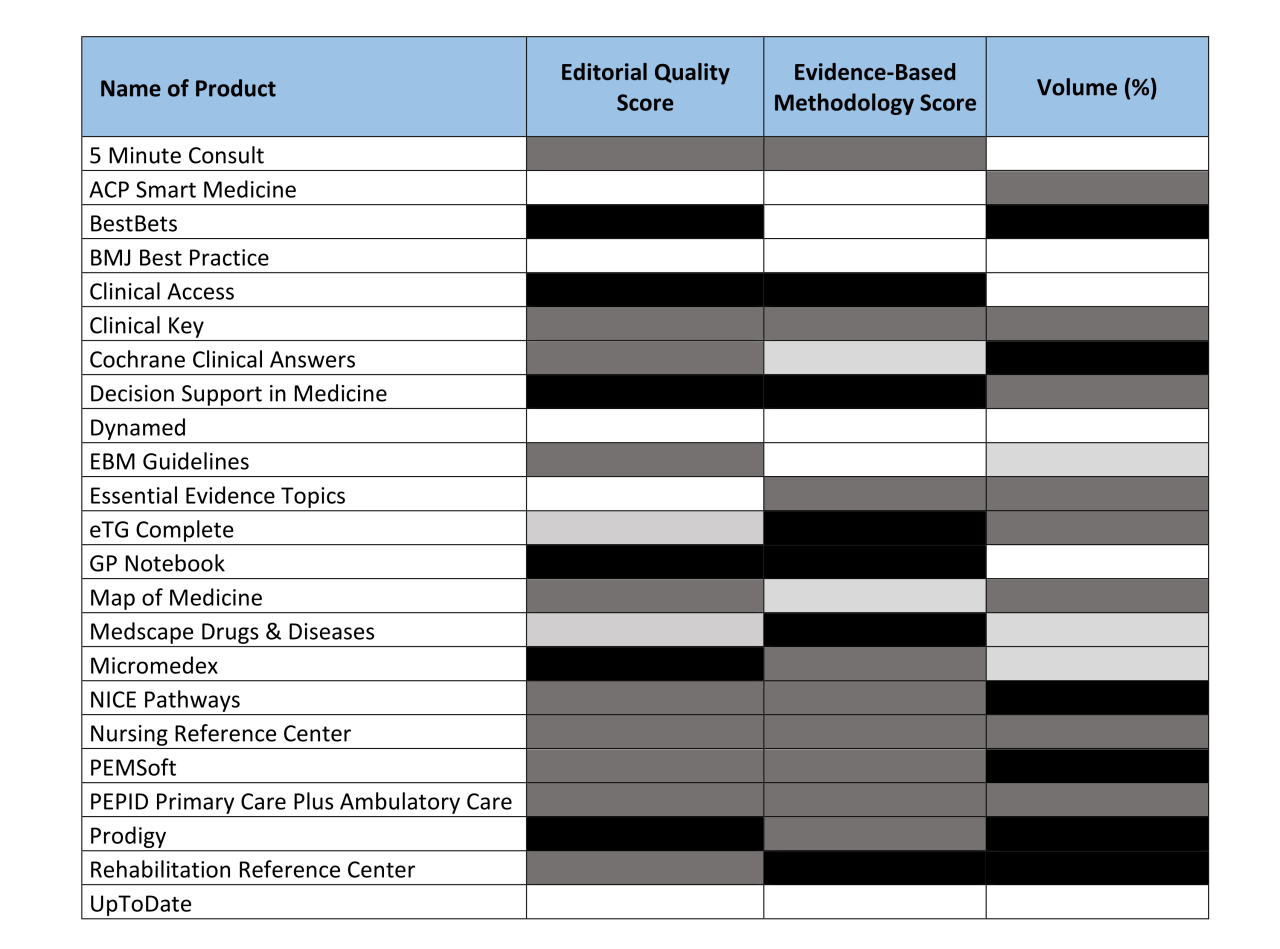
Point-of-care information summary rankings with providers listed in alphabetical order. Quartiles according to 2014 rankings for volume, editorial quality, and evidence-based methodology: black, bottom quartile; dark gray, low intermediate quartile; light gray, high intermediate quartile; white, top quartile (for evidence-based methodology and volume, white represents only the maximum scores of 15 and 100, respectively, as the top quartiles fell on the maximum scores).

The ranking of point-of-care information summaries based on their strength of volume, editorial quality, and evidence-based methodology is shown in Figure 3 (full data reported in Multimedia Appendices 4-6). Best Practice, Dynamed, and UpToDate scored in the highest quartile across all three dimensions. There was a moderate positive correlation between the editorial quality and evidence-based methodology of products (*r*=.41, *P*=.0496). No correlations were found between editorial quality and volume (*r*=.10, *P*=.64), or between evidence-based methodology and volume (*r*=.06, *P*=.80).

**Figure 4:**
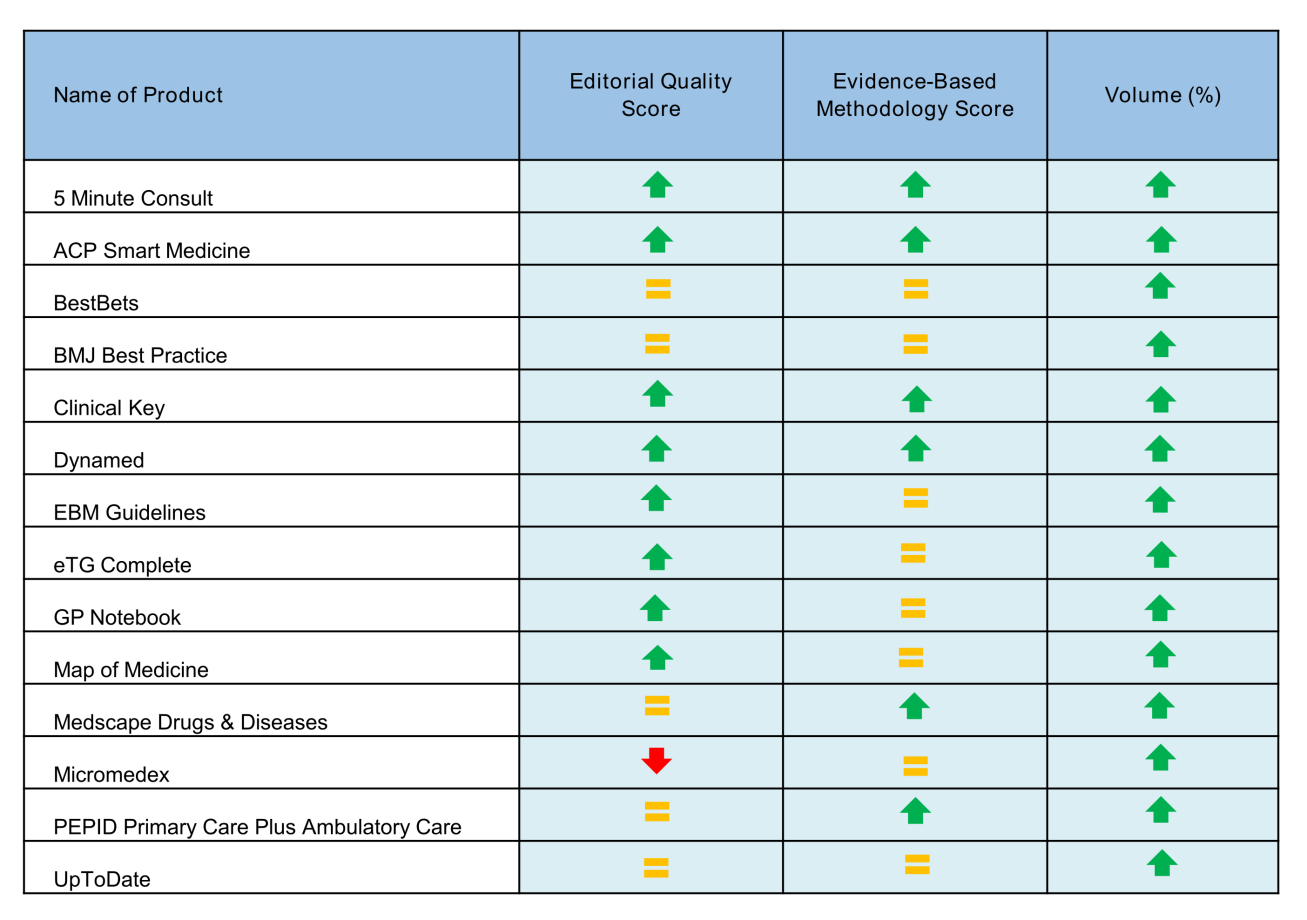
Evolution of the scores for products evaluated in 2008 and reevaluated in 2014. We wrote “=” when the point-of-care information summary score did not change over time, “↑” when the score improved, and “↓” when the score decreased.

Compared to the 2008 evaluation, there were significant improvements in all three dimensions: editorial quality (*P*=.01), evidence-based methodology (*P*=.015), and volume (*P*<.001). Figure 4 shows the evolution of the products in the 2014 assessment that were previously evaluated in 2008.

## Discussion

### Principal Findings

To evaluate products’ claims to be evidence-based, we adopted editorial policy, content quality, and coverage of medical knowledge as the key indicators of high-quality point-of-care information summaries. In line with the 2008 and 2012 analyses, the purpose of our study was not to pinpoint the “winning” and “losing” products but to assess the maturity of these tools for clinical decision making and encourage transparent reporting of editorial and content development policies by publishers. We further sought to guide readers in the selection of products for individual or institutional use. Since 2008, there have been improvements in the general features of point-of-care information summaries and the descriptions of their editorial approaches, though suboptimal products are still on the market [11].

Several limitations to our study must be noted, including use of editorial policy statements to determine the implementation of a formal and structured peer-review process and the acceptance of commercial support for content development. We acknowledge that there may have been discrepancies between the reporting and actual implementation of editorial policies. Moreover, although we included quality dimensions informed by evidence in our study, our criteria for assessment may be perceived as arbitrary; users of a given point-of-care summary may have different views or experience. Regardless of potential differences in opinions, one observation remains clear: publishers have invested notable energy and resources to raise their quality standards in a limited time. Product maturity and the increasing value of reliable information in medical society may sustain the rising popularity of point-of-care summaries among health professionals.

A particular challenge within our study involved the defining of the intervention and execution of the search strategy to identify relevant interventions for inclusion. Since our first evaluation in 2008, there continues to be a discrepancy in the terminology adopted to describe what we identify as “point of care information summaries”: Web-based medical compendia that are specifically designed to deliver predigested, rapidly accessible, comprehensive, and periodically updated information to health care providers. These products have been additionally referred to as “evidence-based textbooks” [22], “clinical point-of-care tools” [23], navigators, and services [3]. While we recognize that other terms might be used, we have adopted point-of-care information summaries as the preferred terminology, as it embraces several key content elements. Given the rising interest and adoption of these tools, the development of a common term and definition will facilitate their assessment by researchers as well as by hospitals and health care professionals in search of a compatible tool for use. A common definition might also benefit the PubMed MeSH vocabulary. In fact, the MeSH term “point-of-care systems” comprises a broad range of health care technologies outside of our intervention, such as laboratory and diagnostic instruments [24].

The quality of most products is still moderate, which has also been indicated by the few additional surveys evaluating the quality of point-of-care information summaries [22,25-27]. Clinicians should become familiar with the basic concepts that make an information product a credible source of scientific evidence. Health libraries and local knowledge brokers should endorse and give preference to summaries that are committed to policies to improve editorial and methodological rigor, disclose conflicts of interest [28-30], and ensure complete and accessible reporting of the content development procedure. Users should be skeptical about point-of-care summaries that do not transparently describe how information is found (search strategy), selected (cumulative or discretionary approach), evaluated (critical appraisal), prioritized (grading of evidence and recommendations), and regularly updated (literature surveillance) to maintain their relevance to practice. Publishers may be highly skilled in boosting clinical recommendations through propaganda and legally qualified to sell their products to doctors and hospitals. Moreover, the failure to disclose methods for product development is not in the best interests of the medical community, and might, in fact, draw the line between authoritative and fraudulent therapeutic information.

Point-of-care information summaries largely serve high-income countries. However, information on highly effective medicines and interventions are presumably more valuable in low- and middle-income countries. At the same time, in an increasingly competitive market, publishers cannot make the service “free for everyone” because this would affect their sustainability and might facilitate the opportunistic use of these resources. We encourage publishers to align the prices of their products to the purchasing power of a particular country’s physicians through tiered-pricing models and to distribute access through networks active in low- and middle-income countries [31,32]. In addition to their affordability and access, the source of information is critical to the strength and reliability of products.

Dynamed currently has links to over 17,000 guidelines, organized for high or low- to middle-income countries [33]. While the consideration of ready-to-use recommendations is a key first step, more investments in tailoring information to local doctors and other health care providers are needed. For example, information on medicines was never ranked on the basis of the WHO Model List of Essential Medicines, which selects treatments that offer a cure or effective disease management in preference to those that offer only marginal benefit [34]. Doctors are increasingly interested in knowing potential incongruence between investing resources and desired health outcomes [35,36]. In this time of austerity, point-of-care summaries have to do a better job considering the social proven value of medicines.

### Future Considerations

It is not easy to predict what directions publishers should take to further improve their services. We propose three approaches. First, as summary providers mature and their contents become broader and more complete (eg, information about medicines, recommendations, and guidelines), information must be re-filtered to meet personal practice needs. Users will need to personalize the product, setting filters to isolate specific information (eg, local hospital guidelines) that is relevant to individual clinical practice. This will prioritize information that can engender changes in health professional behavior [37].

Second, high-quality point-of-care summaries should be integrated into computer decision support systems for EHRs. These computer systems may represent the future of clinical decision making in which evidence-based knowledge from point-of-care summaries is linked with patient information from EHRs to generate case-specific guidance messages through rule- or algorithm-based software [3,38]. Computer decision support systems combined with EHRs might be beneficial for the health care provided to patients, although it is hard to demonstrate their association with benefits on outcomes such as mortality [39].

Third, the potential integration of point-of-care summaries into continuing medical education programs should be recognized [40]. Doubts that are raised during clinical consultation can trigger point-of-care searches that provide health professionals with valuable information that can be directly implemented in the visit. Accreditation systems need to recognize the role of point-of-care summaries as an efficient provider of relevant knowledge.

### Conclusion

The maturation of point-of-care summaries can be seen as a virtuous circle [41]. It started with an exogenous factor: technological innovation. As health professionals become increasingly familiar with the summaries, their adoption will become self-reinforcing. In a competitive market, this will probably help lower product prices, leading to more potential users. The last 20 years saw the success of PubMed, The Cochrane Library, and, more recently, WikiProject Medicine, which are now integral parts of medical practice. Publishers and developers of point-of-care summaries need to direct their considerable talents and resources to developing strategies to sustain affordable practice and interventions to improve quality of practice. This change of focus can support their development as indispensable professional tools.

## Appendix

Multimedia Appendix 1 Flow diagram of methods used to evaluate editorial quality, evidence-based methodology, and volume.

Multimedia Appendix 2 General characteristics of point-of-care information summaries.

Multimedia Appendix 3 Content presentation of point-of-care information summaries.

Multimedia Appendix 4 Editorial quality of point-of-care information summaries.

Multimedia Appendix 5 Evidence-based methodology of point-of-care information summaries.

Multimedia Appendix 6 Point-of-care information summary scores for editorial quality, evidence-based methodology, and volume.

## Abbreviations

EHR: electronic health record
ICD-10: International Classification of Diseases, 10th Revision
IQR: interquartile ranges
