# Supplementary material for: Providing Doctors With High-Quality Information: An Updated Evaluation of Web-Based Point-of-Care Information Summaries
Source: J Med Internet Res. 2016 Jan 19;18(1):e15. doi: 10.2196/jmir.5234 (PMC4738183; doi:10.2196/jmir.5234)

Multimedia Appendix 1. Flow diagram of methods used to evaluate editorial quality, evidence-based methodology and volume

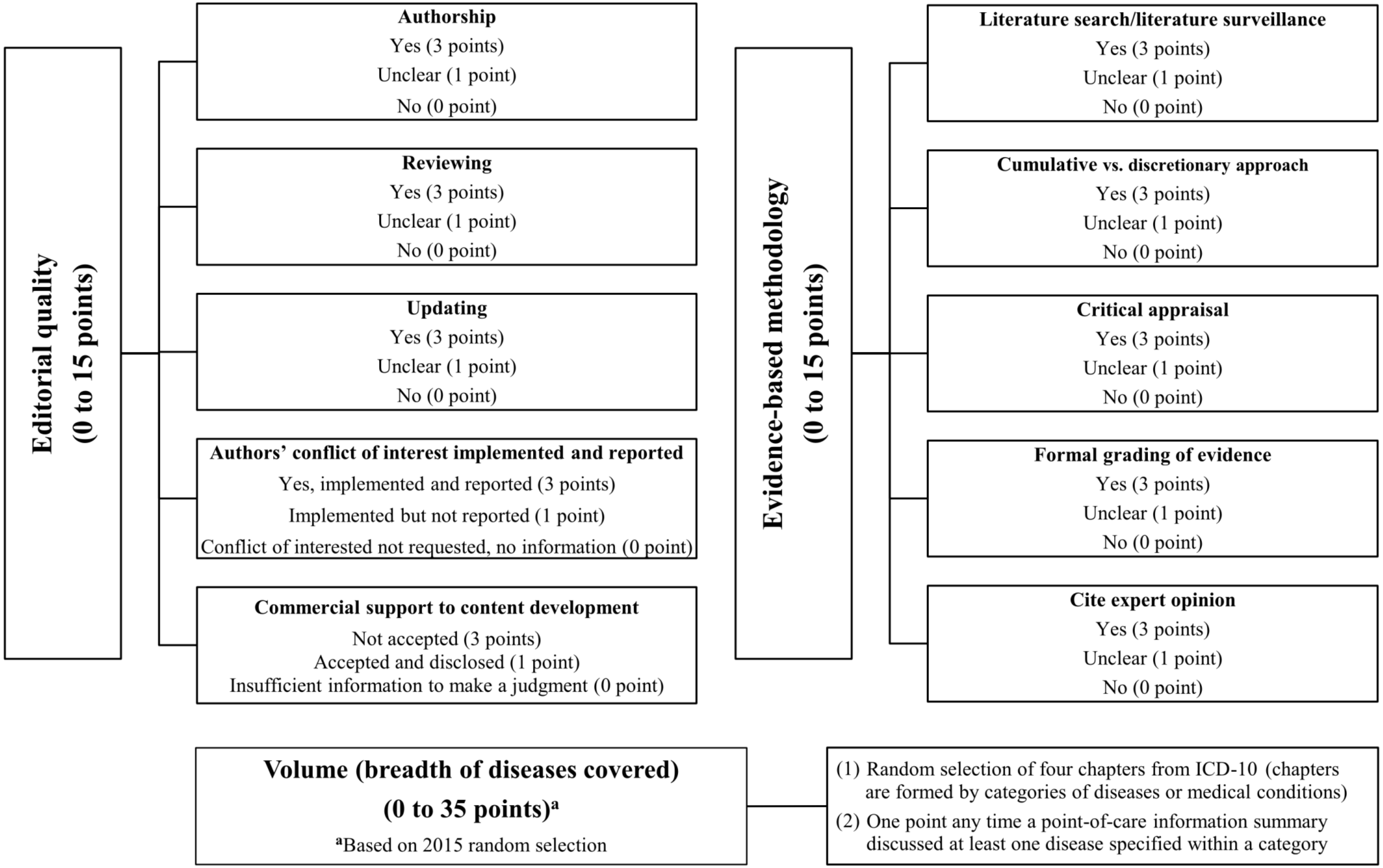

Supplement: Multimedia Appendix 1 [file jmir_v18i1e15_app1.pdf]
