# Supplementary material for: Providing Doctors With High-Quality Information: An Updated Evaluation of Web-Based Point-of-Care Information Summaries
Source: J Med Internet Res. 2016 Jan 19;18(1):e15. doi: 10.2196/jmir.5234 (PMC4738183; doi:10.2196/jmir.5234)
Supplement: Multimedia Appendix 2 [file jmir_v18i1e15_app2.pdf]

Multimedia Appendix 2. General Characteristics of point-of-care information summaries.

Product Name (URL)	Vendor/Publisher	Country	Year of release	Marketing claim	Access	Type of subscription	Format desktops, tablets, and mobile devices	EHR integration	Annual cost (individual subscription)	Target audience
5 Minute Consult (https://5minuteconsult.com)	Wolters Kluwer	Netherlands	not reported	Five minutes is all you need	Fee-based	individual or institutional	online and mobile devices[a]	yes	$99	physicians and healthcare professionals
ACP Smart Medicine (ACP Pier) (http://smartmedicine.acponline.org)	American College of Physicians	USA	2013	One Click to Confidence	fee-based, open access to ACP members	individual	online and mobile devices[a]	no	$265	healthcare professionals
BestBets (http://www.bestbets.org)	Department of Emergency Medicine, Manchester Royal Infirmary	UK	1996	Best evidence topics	open access	n/a	online and print	no	n/a	emergency medicine specialists
BMJ Best Practice (Clinical Evidence) (http://bestpractice.bmj.com)	British Medical Journal Publishing Group Ltd.	UK	2009	Your instant second opinion	fee-based	individual or institutional	online and mobile devices[b]	yes	£142 / €192 / $277 (plus VAT if applicable)	healthcare professionals (and medical students)
Clin-eguide (http://clineguide.ovid.com)	Wolters Kluwer and Unbound Medicine	Netherlands	not reported	Evidence-based and synoptic diagnostic and treatment recommendations for frequent and high-cost problems occurring in primary care, inpatient, and emergency department settings.	fee-based	not reported	online and mobile devices[b]	yes	not reported	healthcare professionals
Clinical Access (http://clinicalaccess.mhmedical.com) [c]	McGraw-Hill Global Education Holdings, LLC.	USA	2014	Targeted answers. Better patient care.	fee-based	individual or institutional	online and mobile devices[a]	no	$395	physicians, residents, nurse practitioners, and physician assistants
Clinical Key (First Consult) (https://www.clinicalkey.com)	Elsevier Inc.	Netherlands	2012	Lead with answers.	fee-based	individual or institutional	online and mobile devices[a]	yes	specialty packages from $449 to 1248	healthcare executives, physicians, nurses, medical librarians, and medical students
Cochrane Clinical Answers (http://cochraneclinicalanswers.com)	Cochrane Innovations Ltd. and John Wiley & Sons, Ltd.	UK	2014	Accessible, authoritative, independent	fee-based	individual or institutional	online	no	contact for pricing	healthcare practitioners and professionals, and other informed health care decision-makers

Decision Support in Medicine (https://www.decisionsupportinmedicine.com)	Decision Support in Medicine, LLC. and Swets	USA	2013	Only the vitals at the point of care	fee-based	individual	online and mobile devices[a]	no	$100 per specialty program	medical specialists
Dynamed (https://dynamed.ebscohost.com)	EBSCO Industries, Inc.	USA	not reported	The Dynamed Difference: DynaMed™ is unique among clinical information resources.	fee-based	individual or institutional	online and mobile devices[b]	yes	$99.95 - $395 depending on profession	physicians and other healthcare professionals
EBM Guidelines (https://www.essentialevidenceplus.com)	Wiley Blackwell Interscience and Duodecim	Finland	1989	Easy to use clinical guidelines supported by sound scientific evidence - an essential tool for the fast pace of primary care	fee-based	individual or institutional	online and mobile devices[a]	yes	$85 (cost for Essential Evidence Plus subscription)	healthcare professionals (targets primary care, ambulatory care and community hospitals, according to NHS accreditation)
Essential Evidence Topics (https://www.essentialevidenceplus.com)	John Wiley & Sons, Inc.	UK	2008	The cornerstone medical reference providing highly relevant, trusted content in a concise, structured evidenced-based format.	fee-based	individual or institutional	online and mobile devices[a]	yes	$85 (cost for Essential Evidence Plus subscription)	physicians, nurses, and other health care professionals
eTG Complete (http://www.tg.org.au)	Therapeutic Guidelines Ltd. (Australia)	Australia	1978	Improving health outcomes through authoritative, practical, independent therapeutic guidance	fee-based	individual or institutional	online, mobile devices[a], CD, and print	no	AUD$335 (full price); AUD$299 (students)	prescribers (general practitioners and trainee physicians in particular)
GP Notebook (https://www.gpnotebook.co.uk; www.univadis.co.uk)	Oxbridge Solutions Ltd.	UK	1995	a UK medical reference	open access for health care professionals through Univadis®, students and academics, and developing countries; fee-based to all others	n/a	online and mobile devices[b]	no	n/a	general practitioners
Map of Medicine (http://mapofmedicine.com)	Hearst Corporation	UK	2001	Better decision making, better care.	fee-based	institutional	online	yes	contact for pricing	medical professionals
Medscape Drugs & Diseases (Emedicine) (http://reference.medscape.com)	WebMD LLC.	USA	not reported	Medscape's clinical reference is the most authoritative and accessible point-of-care medical reference for physicians and healthcare professionals, available online and via all major mobile devices.	open access	n/a	online and mobile devices[b]	no	n/a	physicians and other healthcare professionals

Micromedex (with following subscriptions: Alternative Medicine, DISEASEDEX™ Emergency Medicine, DRUGDEX® System, Imprint Codes in Identidex®, Interaction Checking, Italian Drug Database) (http://micromedex.com)	Truven Health Analytics	USA	not reported	Trusted Evidence for Confident Clinical Decisions	fee-based	not reported	online and mobile devices[b]	yes	contact for pricing	clinicians
Mosby's Nursing Consult (http://www.nursingconsult.com)	Elsevier Inc.	Netherlands	2006	Provide the fast, accurate clinical information nurses need.	fee-based	not reported	online and mobile devices[b]	yes	contact for pricing	nurses
NICE Pathways (http://pathways.nice.org.uk)	National Institute for Health and Care Excellence (NICE)	UK	not reported	Mapping our guidance	open access	n/a	online	no	n/a	users of NICE guidance, including health and social care professionals, public health experts, those who commission or provide health and social care services, employers, and members of the public
Nursing Reference Center (http://www.ebscohost.com/nursing/products/nursing-reference-center)	EBSCO Industries, Inc.	USA	not reported	Get the Most Current Information About Nursing Best Practices	fee-based	individual or institutional	online and mobile devices[b]	yes	contact for pricing	practicing nurses, nursing administrators, and nursing faculty
PEMSoft (http://health.ebsco.com/products/pemsoft/clinical-decision-support)	EBSCO Industries, Inc.	USA	2003 (acquired by EBSCO in 2013)	The Definitive Pediatric Resource for Clinical Decision Support at the Point of Care.	fee-based	individual or institutional	online and mobile devices[b]	Yes	contact for pricing	pediatricians and other medical specialists working with neonatal, infant, child, adolescent and young adult health
PEPID Primary Care Plus Ambulatory Care (http://www.pepid.com)	PEPID, LLC.	USA	1994	PEPID Primary Care Plus is a comprehensive, integrated reference tool covering all medical specialties. PCP enables you to bring all the information you need to the bedside – improving quality, safety and efficiency of	fee-based	individual or institutional	online and mobile devices[b]	yes	US$299.95	physicians, physician assistants, nurse practitioners, and residents

				care.						
Prodigy (NICE Clinical Knowledge Summaries) (http://prodigy.clarity.co.uk)	Clarity Informatics (Prodigy); National Institute for Health and Care Excellence (NICE) (CKS accessed within UK)	UK	CKS available since 1998	Clinical guidance for common conditions and symptoms presenting in primary care	open access to UK users, fee-based for non-UK users	individual or institutional	online	no	£100 (full price); £50 (students)	primary care physicians
Rehabilitation Reference Center (http://health.ebsco.com/products/rehabilitation-reference-center)	EBSCO Industries, Inc.	USA	2008	Get the Most Current Information About Sports Medicine and Rehabilitation Best Practices	fee-based	individual or institutional	online	yes	contact for pricing	physical therapists, occupational therapists, speech therapists, and rehabilitation professionals.
UpToDate (http://www.uptodate.com)	UpToDate, Inc. (Wolters Kluwer)	USA	1992	Smarter Decisions. Better Care.	fee-based	individual or institutional	online and mobile devices[b]	yes	US$199	healthcare practitioners
ZynxEvidence (http://www.zynxhealth.com)	Zynx Health, Inc. (Hearst Corporation)	USA	not reported	Online Clinical Evidence Foundation	fee-based	not reported	online	yes	not reported	clinicians

[a] Point of care information summary interface adapts to mobile screens
[b] Separate mobile version or application available for download
[c] Contents based on several sources of information, including Harrison's Practice that was evaluated in 2008.
