# Supplementary material for: Providing Doctors With High-Quality Information: An Updated Evaluation of Web-Based Point-of-Care Information Summaries
Source: J Med Internet Res. 2016 Jan 19;18(1):e15. doi: 10.2196/jmir.5234 (PMC4738183; doi:10.2196/jmir.5234)
Supplement: Multimedia Appendix 3 [file jmir_v18i1e15_app3.pdf]

Multimedia Appendix 3. Content Presentation of point-of-care summaries.

Name	Type of output	Formal ontology	Summary flexibility	References	Intent to recommend	Strength of recommendation formal system	CME programs	Other educational materials (topics)	Patient handouts
5 Minute Consult	key point summary	yes	yes	yes, specific	yes	yes[a]	yes	no	yes
ACP Smart Medicine	key point summary	yes	yes	yes, specific	yes	no	yes	no	no
Best Bets	question and answer, evidence tables	yes	no	yes, specific	no	no	no	yes	no
BMJ Best Practice	key point summary, paragraphs, question and answer, evidence tables	yes	yes	yes, specific	yes	yes	yes	no	yes
Clinical Access	question and answer, paragraphs	yes (no link)	yes	yes, specific	no	no	no	no	no
Clinical Key	key point summary	yes	yes	yes, specific	yes	no	yes	no	yes
Cochrane Clinical Answers	question and answer	yes	yes	yes, specific	no	no	no	no	no
Decision Support in Medicine	question and answer, paragraphs, key point summary	yes	yes	yes, general	yes	no	no	no	no
Dynamed	key point summary	yes	yes	yes, specific	yes	yes	yes	no	no
EBM Guidelines	key point summary	yes	no	yes, general and specific	yes	no	no	no	no
Essential Evidence Topics	key point summary	yes	yes	yes, specific	yes	yes[a]	yes	no	no
eTG Complete	book chapter- like summary, key point summary	yes	yes	yes, general	yes	no	no	no	no
GP Notebook	book chapter- like summary, key point summary	yes	no	yes, general	no	no	yes	no	no
Map of Medicine	clinical pathways	yes	yes	yes, specific	yes	no	no	no	no
Medscape Drugs & Diseases	book chapter- like summary, key point summary	yes	no	yes, general	yes	no	yes	no	no
Micromedex	book chapter- like summary, key point summary, evidence tables	no	yes	yes, specific	yes	yes	no	no	no

NICE Pathways	clinical pathways with key point summary and visual representation	yes	yes	yes, general	yes	no	no	no	yes
Nursing Reference Center	book chapter- like summary, key point summary	no	yes	yes, general and specific	yes	no	yes	yes	yes
PEMSoft	key point summary	yes	yes	yes, general	yes	no	no	no	no
PEPID Primary Care Plus Ambulatory Care	key point summary	yes	yes	yes, specific	yes	yes[a]	yes	yes	no
Prodigy	key point summary, clinical scenario	yes	yes	yes, general and specific	yes	no	no	no	no
Rehabilitation Reference Center	key point summary	no	yes	yes, specific	yes	no	no	yes	yes
UpToDate	book chapter- like summary, key point summary	yes	yes	yes, specific	yes	yes	yes	no	yes

[a] SORT strength of recommendation taxonomy used
