# Supplementary material for: Providing Doctors With High-Quality Information: An Updated Evaluation of Web-Based Point-of-Care Information Summaries
Source: J Med Internet Res. 2016 Jan 19;18(1):e15. doi: 10.2196/jmir.5234 (PMC4738183; doi:10.2196/jmir.5234)
Supplement: Multimedia Appendix 4 [file jmir_v18i1e15_app4.pdf]

Multimedia Appendix 4. Editorial quality of point-of-care information summaries.

Name	Authorship	Reviewing	Updating	Authors' conflict of interest implemented and reported	Commercial support to content development	Editorial quality score
5 Minute Consult	yes (3)	no information (0)	unclear (1)	no (0)	not accepted (3)	7
ACP Smart Medicine	yes (3)	yes (3)	yes (3)	yes (3)	not accepted (3)	15
BestBets	yes (3)	yes (3)	no (0)	no (0)	insufficient information to make a judgement (0)	6
BMJ Best Practice	yes (3)	yes (3)	yes (3)	yes (3)	not accepted (3)	15
Clinical Access	no (0)	yes (3)	yes (3)	no (0)	insufficient information to make a judgement (0)	6
Clinical Key	yes (3)	yes (3)	no (0)	no (0)	not accepted (3)	9
Cochrane Clinical Answers	yes (3)	yes (3)	yes (3)	no (0)	not accepted (3)	12
Decision Support in Medicine	yes (3)	unclear (1)	unclear (1)	no (0)	insufficient information to make a judgement (0)	5
Dynamed	yes (3)	yes (3)	yes (3)	yes (3)	not accepted (3)	15
EBM Guidelines	yes (3)	yes (3)	yes (3)	no (0)	not accepted (3)	12
Essential Evidence Topics	yes (3)	yes (3)	yes (3)	yes (3)	not accepted (3)	15
eTG Complete	yes (3)	yes (3)	unclear (1)	yes (3)	not accepted (3)	13
GP Notebook	no (0)	unclear (1)	unclear (1)	yes (3)	insufficient information to make a judgement (0)	5
Map of Medicine	no (0)	yes (3)	yes (3)	no (0)	not accepted (3)	9
Medscape Drugs & Diseases	yes (3)	yes (3)	yes (3)	yes (3)	accepted and disclosed (1)	13
Micromedex	no (0)	yes (3)	unclear (1)	no (0)	insufficient information to make a judgement (0)	4
NICE Pathways	no (0)	unclear (1)	yes (3)	no (0)	not accepted (3)	7
Nursing Reference Center	yes (3)	yes (3)	yes (3)	no (0)	not accepted (3)	12
PEMSoft	yes (3)	yes (3)	yes (3)	no (0)	not accepted (3)	12

PEPID Primary Care Plus Ambulatory Care	yes (3)	yes (3)	yes (3)	no (0)	insufficient information to make a judgement (0)	9
Prodigy	no (0)	unclear (1)	unclear (1)	no (0)	not accepted (3)	5
Rehabilitation Reference Center	yes (3)	yes (3)	yes (3)	no (0)	not accepted (3)	12
UpToDate	yes (3)	yes (3)	yes (3)	yes (3)	not accepted (3)	15
