# Supplementary material for: Providing Doctors With High-Quality Information: An Updated Evaluation of Web-Based Point-of-Care Information Summaries
Source: J Med Internet Res. 2016 Jan 19;18(1):e15. doi: 10.2196/jmir.5234 (PMC4738183; doi:10.2196/jmir.5234)
Supplement: Multimedia Appendix 5 [file jmir_v18i1e15_app5.pdf]

Multimedia Appendix 5. Evidence-based methodology of point-of-care information summaries

Name	Literature search/literature surveillance	Cumulative vs. discretionary approach	Critical appraisal	Formal grading of evidence	Cite expert opinion	Total score
5 Minute Consult	unclear (1)	unclear (1)	unclear (1)	yes (3)	unclear (1)	7
ACP Smart Medicine	yes (3)	yes (3)	yes (3)	yes (3)	yes (3)	15
BestBets	yes (3)	yes (3)	yes (3)	yes (3)	yes (3)	15
BMJ Best Practice	yes (3)	yes (3)	yes (3)	yes (3)	yes (3)	15
ClinicalAccess	unclear (1)	no (0)	no (0)	no (0)	no (0)	1
Clinical Key (First Consult)	unclear (1)	yes (3)	unclear (1)	yes (3)	yes (3)	11
Cochrane Clinical Answers	unclear (1)	yes (3)	yes (3)	yes (3)	yes (3)	13
Decision Support in Medicine	unclear (1)	unclear (1)	unclear (1)	no (0)	no (0)	3
Dynamed	yes (3)	yes (3)	yes (3)	yes (3)	yes (3)	15
EBM Guidelines	yes (3)	yes (3)	yes (3)	yes (3)	yes (3)	15
Essential Evidence Topics	unclear (1)	yes (3)	yes (3)	yes (3)	unclear (1)	11
eTG Complete	unclear (1)	no (0)	no (0)	no (0)	no (0)	1
GP Notebook	unclear (1)	no (0)	no (0)	no (0)	no (0)	1
Map of Medicine	yes (3)	yes (3)	yes (3)	no (0)	yes (3)	12
Medscape Drugs & Diseases	unclear (1)	no (0)	no (0)	no (0)	unclear (1)	2
Micromedex	yes (3)	unclear (1)	yes (3)	yes (3)	unclear (1)	11
NICE Pathways	unclear (1)	unclear (1)	unclear (1)	unclear (1)	unclear (1)	5
Nursing Reference Center	yes (3)	yes (3)	yes (3)	no (0)	unclear (1)	10

PEMSoft	unclear (1)	unclear (1)	yes (3)	no (0)	no (0)	5
PEPID Primary Care Plus Ambulatory Care	yes (3)	unclear (1)	yes (3)	yes (3)*	unclear (1)	11
Prodigy	yes (3)	yes (3)	unclear (1)	no (0)	unclear (1)	8
Rehabilitation Reference Center	unclear (1)	unclear (1)	unclear (1)	no (0)	unclear (1)	4
UpToDate	yes (3)	yes (3)	yes (3)	yes (3)	yes (3)	15
