# Supplementary material for: Providing Doctors With High-Quality Information: An Updated Evaluation of Web-Based Point-of-Care Information Summaries
Source: J Med Internet Res. 2016 Jan 19;18(1):e15. doi: 10.2196/jmir.5234 (PMC4738183; doi:10.2196/jmir.5234)
Supplement: Multimedia Appendix 6 [file jmir_v18i1e15_app6.pdf]

Multimedia Appendix 6. Point-of-care information summary scores for editorial quality, evidence-based methodology, and volume.

Name of product	Editorial quality score	Evidence-based methodology score	Volume (%)
5 Minute Consult	7	7	100
ACP Smart Medicine	15	15	77
BestBets	6	15	63
BMJ Best Practice	15	15	100
Clinical Access	6	1	100
Clinical Key	9	11	94
Cochrane Clinical Answers	12	13	63
Decision Support in Medicine	5	3	94
Dynamed	15	15	100
EBM Guidelines	12	15	97
Essential Evidence Topics	15	11	94
eTG Complete	13	1	89
GP Notebook	5	1	100
Map of Medicine	9	12	89
Medscape Drugs & Diseases	13	2	97
Micromedex	4	11	97
NICE Pathways	7	5	54
Nursing Reference Center	12	10	91
PEMSoft	12	5	66
PEPID Primary Care Plus Ambulatory Care	9	11	94
Prodigy	5	8	63
Rehabilitation Reference Center	12	4	26
UpToDate	15	15	100
**Median (Interquartile Range)**	**12 (6-13)**	**11 (4-15)**	**94 (66-100)**
